# Dynamics in Endolymphatic Hydrops & Symptoms in Meniere's Disease After Endolymphatic Duct Blockage, Preliminary Results

**DOI:** 10.3389/fneur.2020.622760

**Published:** 2021-01-21

**Authors:** Jun He, Anquan Peng, Junjiao Hu, Zhiwen Zhang, Yichao Chen, Qin Wang, Wei Liu, Huang Chao, Kai Deng, Wenqi Jiang

**Affiliations:** ^1^Department of Otolaryngology-Head and Neck Surgery, The Second Xiangya Hospital, Central South University, Changsha, China; ^2^Department of Radiology, The Second Xiangya Hospital, Central South University, Changsha, China

**Keywords:** endolymphatic hydrops, endolymphatic duct blockage, Ménière's disease, treatment, dynamics, symptoms

## Abstract

**Objective:** The purpose of the present study was to evaluate the dynamics of endolymphatic hydrops (EH) and symptoms in a group of patients who underwent endolymphatic duct blockage (EDB) for treatment of intractable Meniere's Disease (MD), and to explore a metric for verifying the effectiveness of EDB procedure.

**Methods:** A total of 22 patients with intractable MD patients who underwent EDB participated in the present study. EH was visualized using locally enhanced inner ear magnetic resonance imaging (MRI) prior to and following surgery. The vestibular hydrops ratio (VHR) in the second MRI examination was compared with the pre-surgery recordings.

**Results:** Following EDB, 6 patients exhibited complete or partial reversal of EH, complete control of vertigo spells and reported improvement in hearing; 13 patients showed no changes in EH or hearing, but 5 of these patients exhibited complete control of vertigo attacks, and the other 8 patients exhibited improved control of vertigo attacks. The final 3 patients showed an increase in EH, but symptomatic worsening in 2 patients, and symptomatic improvement in 1 patient. There was a significant difference in the average VHR prior to and following EDB. Postoperative VHR was positively correlated with the frequency of vertigo spells in the latest 6 months of follow-up and improvement of postoperative average hearing threshold.

**Conclusion:** The decreased EH accompanying the reduction in vertigo attacks and hearing preservation may provide a metric for verifying the effectiveness of EDB treatment in patients with MD.

## Introduction

Meniere's disease (MD), an idiopathic inner ear disorder, *is characterized by* recurring attacks of vertigo, fluctuating hearing loss, tinnitus, and ear fullness (1). The specific underlying pathophysiological mechanisms of MD are unknown, but treatments continue to focus on controlling the symptoms of MD either through medications or by surgical intervention (2). However, as symptoms are known to fluctuate in MD, the majority of patients notice improvements regardless of the specific therapy used. Placebo effects and failure to consider spontaneous improvement are notable problems of previous studies (3). As a treatment option, endolymphatic sac (ES) surgery, which is based on the hypothesis that deficient absorption in the endolymphatic sac is one of the causes of endolymphatic hydrops (EH), has been extensively used since its introduction by Portman in 1927 (4). However, several studies have shown that endolymphatic sac surgery is no more effective than placebo since the 1980's (5–7). This simply highlights the complexity of this disease and reveals the ongoing needs for further study in optimal treatment options for MD. An immunohistochemical and ultrastructural investigation of the human endolymphatic sac in MD revealed both the secretion of glycoproteins and the possible existence of hypersecretions of endolymph in the sac (8–10), and an increased expression of aquaporin-2 in the endolymphatic sac epithelium of patients with MD was proposed to be involved in the pathophysiology of EH (11), which may support the assumption that an increased secretion outweighs a decreased absorption resulting in increased pressure in the inner ear (12). According to this hypothesis, a novel surgical sac technique, endolymphatic duct blockage (EDB), was proposed for the treatment of MD and shown be effective for the control of symptoms of MD, without any noticeable cochlear and vestibular damage (12). However, the efficacy of this treatment has not been demonstrated, and the underlying mechanisms of its effects are speculative, which is largely due to the lack of an objective marker of treatment effectiveness. As EH is widely recognized as a pathological change in the inner ear associated with MD (13, 14), changes in EH may be used to objectively evaluate the effects of various treatments for patients with MD (15, 16) due to recent advances *in vivo* imaging of EH (17, 18).

In the present study, EDB was used for treatment of a group of patients with MD, prospectively evaluating the changes in EH using gadopentetate dimeglumine (Gd)-enhanced inner ear magnetic ronance imaging (MRI), and the possible associations between symptomatic changes and hydropic changes in the inner ear following surgery were examined, with the aim of exploring a metric for verifying the effectiveness of treatment.

## Materials and Methods

### Patients

According to the 2015 criteria of Classification Committee of the Bárány Society for the diagnosis of MD (19), 88 patients were diagnosed as having definite MD or probable MD from a total of 2,160 patients with vertigo/dizziness who referred to our otology led vertigo clinic between January 2018 and December 2018. Yet, a total of 39 patients with intractable MD were hospitalized and referred for inner ear MRI to demonstrate EH. Twenty-six patients with intractable MD with MRI-based visualization of unilateral EH underwent EDB for treatment of MD at our university hospital. The patients with intractable MD were defined as the patients with recurrent vertigo/dizziness for at least 6 months and failure of systematic medical treatments and psychological management, including appropriate life guidance and oral therapy involving administration of osmotic diuretic medicine and betahistine.

Definitive vertigo/dizziness lasting >20 min was considered a vertigo attack (19). All the patients were asked to keep a daily vertigo diary to document MD episodes. Based on a suggestion by Gu^..^rkov et al. (20) regarding the determining efficacy of control of vertigo in treatment trials by evaluating the number of vertigo attacks during the 6 months before initiation of therapy and during the last 6 months of therapy, the frequency of vertigo spells before EDB was calculated based on the number of vertigo attacks during the 6 months prior to EDB. Frequency of vertigo spells following EDB was calculated based on the number of vertigo attacks during the latest 6 months following EDB.

Hearing function was evaluated by the arithmetic mean of hearing thresholds at 0.5, 1, 2, and 3 kHz.The worst hearing level during the 6 months prior to EDB was used to evaluate the hearing level before surgery, whereas the worst hearing level during the most recent 6 months following surgery was used for evaluation of the hearing level following surgery. Changes in hearing levels were defined: Worse, elevation ≥10 dB; Better, Decline ≥10 dB; And same, −10 dB < x < 0 dB.

After confirmation of EH using gadolinium-enhanced inner ear MRI, and after providing informed consent, the patients underwent surgery. The follow-up was performed between February and April 2020. At follow-up, a second MRI examination was performed and the assessed EH was compared with the pre-surgery examination. A total of 4 patients could not be followed-up for Gd-MRI because they *experienced ear pain* in the first MRI examination and were reluctant to receive second intratympanic injections; 22 subjects met the inclusion criteria (11 females and 11 males, aged 30–65 years, mean age 50.1 years).

The present study complied with the principles of Declaration of Helsinki was approved by the Ethics Committee of the Second Xiangya Hospital (certificate number: S452; the day of the approval: November 22, 2017).

### Surgical Technique

**Figure 1:**
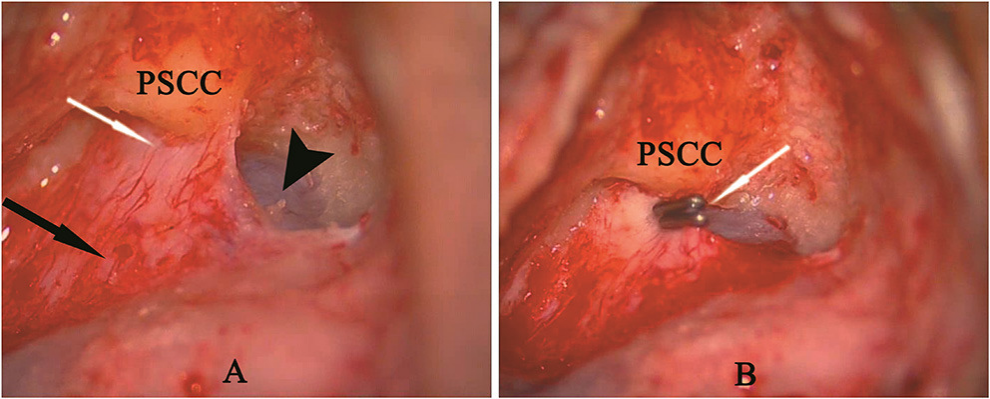
**(A)** A simple mastoidectomy and endolymphatic sac dissection in left ear, showing the intraosseous portion of sac (white arrow), extraosseous portion of sac (black arrow) and posterior fossa dura (arrow head). **(B)** Two titanium clips (white arrow) blocking the endolymphatic duct behind the posterior semicircular canal (PSCC).

The surgical procedure used for EDB was similar to that previously described by Saliba (12). Briefly, a simple mastoidectomy was performed to expose the endolymphatic sac in the area between the sigmoid sinus and the inferior margin of the posterior semicircular canal, including the rugose portion. A pair of small titanium clips was used to block the dissected endolymphatic sac by the ligating clip applier (Figure 1). Postoperative wound management and postoperative care were similar to those used in other mastoid surgical operations.

### Gd-MRI

MRI was performed using intratympanic and intravenous application of gadopentetate dimeglumine (IT-Gd +IV-Gd) as previously described (21). In brief, MRI was performed using a single-dose (0.2 ml/kg) intravenous administration of contrast media (Magnevist®, Bayer AG) 4 h before the MRI scan, as well as intratympanic administration of 8-fold-diluted Gd in both ears 24 h prior to the MRI scan. The off-label use of IT-Gd-MRI was performed under informed consent. For the second Gd-MRI, if patients were reluctant to receive intratympanic injections in their healthy ear, the intratympanic administration of contrast agent was used in the ear undergoing surgery alone. The MRI scan was performed with a three-dimensional real inversion recovery (3D-real IR) sequence on a 3 T MR unit (Magnetom Verio; Siemens AG) using a 12-channel head coil, as previously described (17, 18). Briefly, the parameters for the 3D-real IR sequence were: Voxel size, 0.4 × 0.4 × 0.8 mm; scan time, 14 min; repetition time (TR), 9,000 msec, echo time (TE), 181 msec; inversion time (TI), 1,730 msec; slice thickness, 0.80 mm; field of view (FOV), 160 × 160 mm; matrix size, 3,300 × 918.

### Image Evaluation

**Figure 2:**
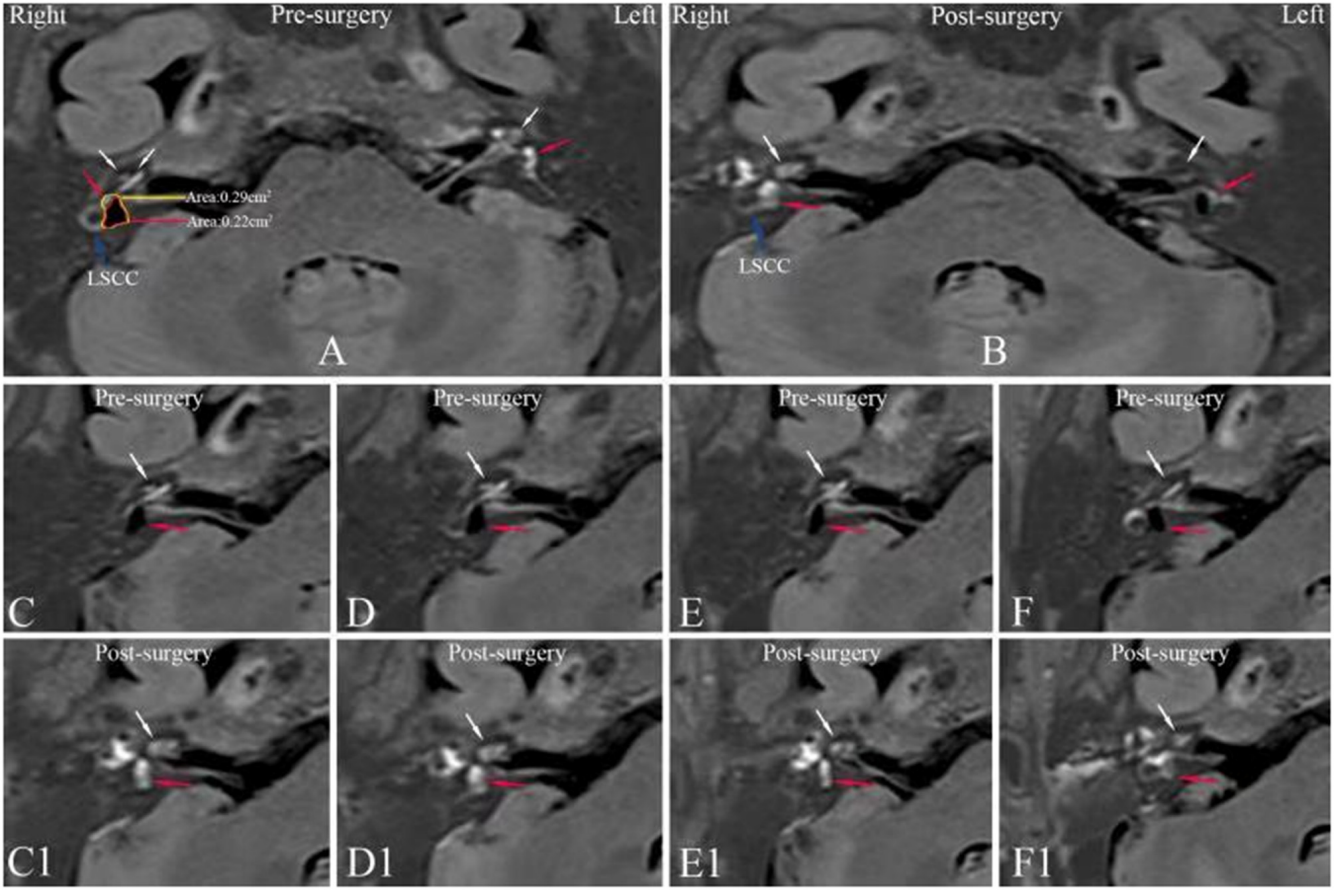
Axial MR images of patient no. 4 (Table 1) with right MD prior to surgery **(A,C–F)** and 20 months after surgery **(B,C1–F1)**. **(A)** 3D-real IR MRI revealing a mild cochlear EH (white arrow) and a significant vestibular EH (red arrow) where the vestibular hydrops ratio is 76% in the right ear and no pathological finding in the left ear. **(B)** In the same slice level as in **(A)**, an axial IT-Gd+IV-Gd MR image of the right affected ear shows bright enhanced perilymphatic fluid in the vestibule (red arrow) and cochlea (white arrow), instead of black non-enhanced area shown in **(A)**, whereas an axial IV-Gd MR image of the left unaffected ear shows insufficient contrast in the inner ear compared with IT-Gd+IV-Gd MR image in **(A)**. Preoperative serial MRIs **(C–F)** show a mild cochlear EH (white arrow) and a significant vestibular EH (red arrow) in right affected ear. Postoperative serial MRIs **(C1–F1)** show a disappearance of EH in the vestibule (red arrow) and cochlea (white arrow). LSCC, lateral semicircular canal; 3D-real IR, three-dimensional real inversion recovery; MRI, magnetic resonance imaging; MD, Meniere's disease; EH, endolymphatic hydrops; IT-Gd, intratympanic injection of gadopentetate dimeglumine; IV-Gd, intravenous injection of gadopentetate dimeglumine.

The degree of EH in cochlea was *classified* as none, mild and significant using 3D-real IR sequence MRI images as described previously by Nakashima (22). In order to reduce test-retest variability between the first image and second image, the axial slices where the lateral semicircular canal ring was visualized >240° were selected for evaluating EH in vestibule (Figure 2A). The proportion of *the endolymphatic and perilymphatic spaces* in the vestibule was calculated using a Siemens Syngo Via workstation based on the outlines of the area traced on the axial images, excluding the semicircular canals and the ampulla. The vestibular hydrops ratio (VHR) was defined as the ratio of the area (%) of the endolymphatic space in the entire lymphatic space in the vestibule, which was calculated as follows: VHR% = [number of negative pixels for the endolymph in the region of interest (ROI)/ total number of pixels in the ROI] × 100. Exemplary MRI image for the *calculation of* VHR is shown in Figure 2A. If the endolymphatic area of the vestibule was >50% of the total vestibular area, it was described as significant endolymphatic hydrops. If the area was ≤1/3 the total area, there was no endolymphatic hydrops. A mild designation was considered any proportion between no and significant endolymphatic hydrops as previously described (22). The evaluation of the vestibular hydrops was based on a suggestion by Uno et al. (23) regarding the determining no change of preoperative and postoperative images when the proportion of the endolymphatic space in vestibular areas was changed by no more than 10 points (%). Increased vestibular hydrops was defined as a VHR which increased >10%. Reduced vestibular hydrops was defined as a VHR which decreased >10%. No change in vestibular hydrops was defined as a VHR change < |10%|. The complete reversal of vestibular hydrops was defined as a VHR ≤ 33.3%.

### Statistical Analysis

For statistical analysis, a paired Student's *t*-test was used for two-group comparisons, whereas Pearson's test was adopted for evaluating the relationship. *p*-values lower than 0.05 were considered significant. All data were statistically treated with SPSS version 26.0 (IBM Corp., Armonk, NY).

## Results

Table 1 shows the clinical profiles before and after surgery including the frequency of vertigo attacks and the dB hearing levels in the corresponding periods and IT-Gd+IV-Gd MRI results. Before surgery, all 22 patients with intractable unilateral MD showed EH in both the cochlea and vestibule in the affected ear, whereas T2-weighted cisternography sequence was used to rule out vestibular schwannoma or endolymphatic sac tumor.

**Table 1:** Preoperative and postoperative clinical profiles.

**Case**	**Age/**	**Hearing(dB)**		**Vertigo attacks**		**Vestibular**		**Cochlear**		**TSM**	**Follow-up**
**no**.	**gender**			**(a/mo)**		**hydrops rate (%)**		**hydrops**			
		**Pre**	**Post**	**Pre**	**Post**	**First**	**Second**	**First**	**Second**	**(Mon)**	**(Mon)**
1	65/M	53.8	52.5	4	0.7	42	45	1	1	22	22
2	51/M	51.3	45	3.3	0	68	62	1	1	22	22
3	37/M	53.8	47.5	4.2	1.8	58	60	1	1	21	21
4	39/F	42.5	27.5	3.8	0	76	14	1	0	20	20
5	50/F	70	40	4.2	0	90	28	2	1	20	20
6	61/F	57.5	62.5	3.8	0.7	48	50	1	1	19	19
7	47/F	42.5	41.3	5.5	1	52	51	1	1	19	19
8	52/F	41.3	57.5	3	3.3	39	50	1	2	18	18
9	65/F	68.8	72.5	3.3	1.2	71	83	2	2	18	18
10	46/M	58.8	38.8	2.5	0	84	48	2	2	18	18
11	64/F	67.5	65	1.7	0.7	62	63	2	2	17	17
12	30/F	63.8	33.8	2.3	0	48	11	1	0	17	17
13	48/M	52.5	56.3	2.5	0	52	45	1	1	16	16
14	62/F	48.8	21.3	4.3	0	74	18	2	1	16	16
15	54/M	67.5	71.3	2.5	1.8	78	79	2	2	15	15
16	48/F	60	33.8	3	0	82	47	2	2	15	15
17	48/M	42.5	45	3.7	1.7	42	44	1	1	15	15
18	52/M	62.5	60	2.5	0	68	65	2	2	14	14
19	60/M	71.3	72.5	3.7	1	56	50	2	2	14	14
20	39/M	38.8	43.8	3.7	4.2	48	72	1	2	14	14
21	49/F	45	46.3	3.3	0	52	49	1	1	13	13
22	36/F	31.3	36.3	3.2	0	39	40	1	1	13	13

*M, Male; F, Female; Mon, Month; a/mo, attack/month; TSM, Time intervals of second MRI examinations following EDB; 0, no hydrops; 1, mild hydrops; 2, significant hydrops*.

### Dynamic Changes of EH on IT-Gd + IV-Gd MRI Prior to and Following Surgery

According to MRI evaluation, the grade of the cochlear EH in 22 patients was subjectively rated as: a mild EH in 13 ears and a significant EH in 9 ears prior to surgery; no hydrops in 2 ears, a mild EH in 11 ears and a significant EH in 9 ears following surgery. Based on the VHR results, the vestibular EH in 22 patients was classified as: a mild EH in 7 ears and a significant EH in 15 ears prior to surgery; no hydrops in 4 ears, a mild EH in 10 ears and a significant EH in 8 ears following surgery.

**Figure 3:**
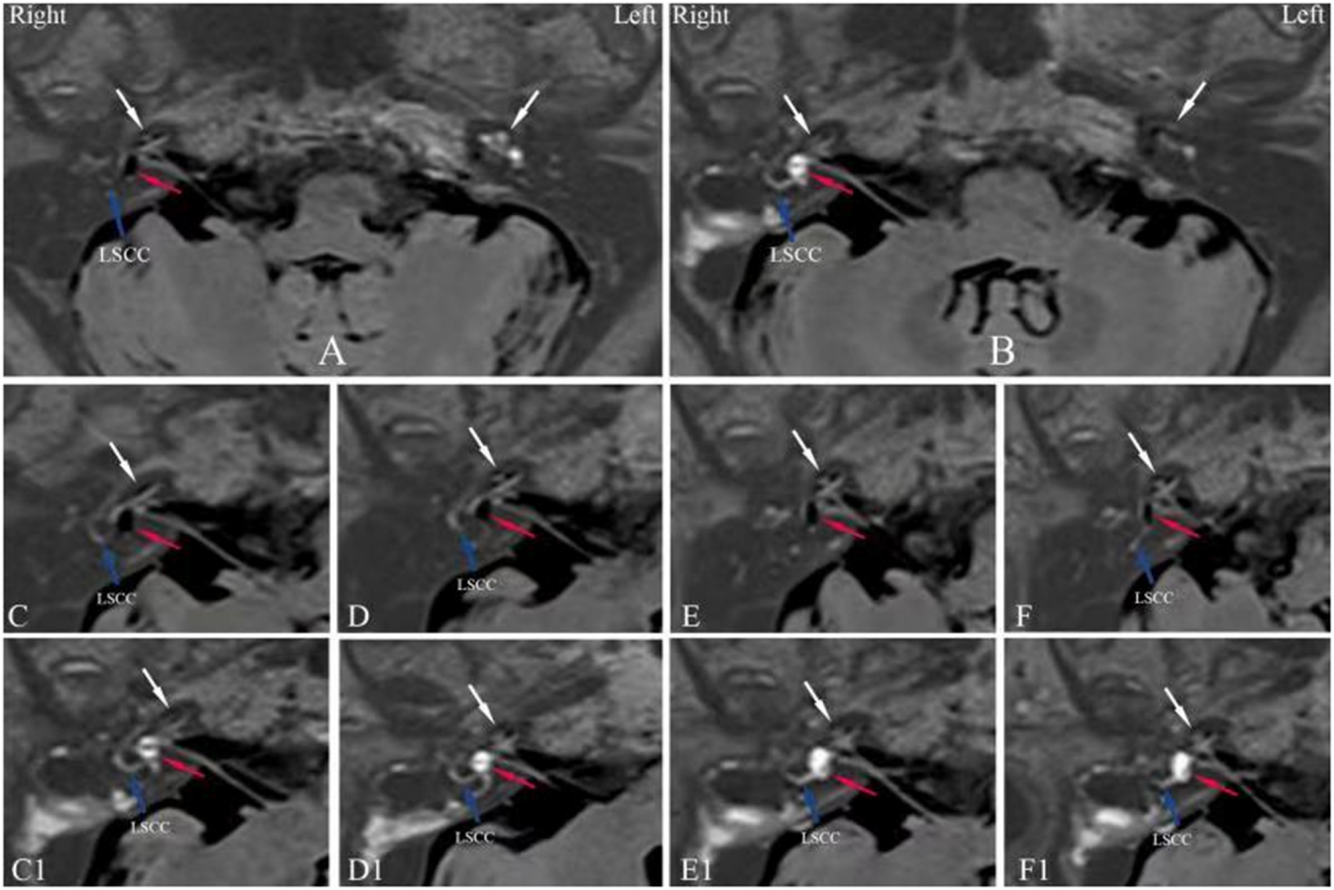
Axial IT-Gd+IV-Gd MRIs of patient no. 14 (Table 1) with right MD prior to surgery **(A,C–F)** and 16 months **(B,C1–F1)** after surgery. **(A)** An significant EH in the vestibule (red arrow) and cochlea (white arrow) in right ear and no signal void (endolymphatic space) with only enhanced perilymphatic space in left unaffected ear (white arrow). **(B)** The image shows complete reversal of vestibular hydrops and partial reversal of cochlear hydrops in the right ear in the same section plane as in **(A)**, where the low-signal area of the vestibule shown in **(A)** has changed to a high-signal area (red arrow) and a significant cochlear EH has changed to a mild EH (white arrow). Whereas, an axial IV-Gd MR image of the left unaffected ear shows insufficient contrast in cochlea (white arrow). The serial MR images in **(C–F)** show a significant EH in vestibule (red arrow) and cochlea (white arrow) before surgery. The serial MR images in **(C1–F1)** show a disappearance of vestibular EH (red arrow) and the reduction of cochlear EH (white arrow) 16 months after surgery. LSCC, lateral semicircular canal; 3D-real IR, three-dimensional real inversion recovery; MRI, magnetic resonance imaging; MD, Meniere's disease; EH, endolymphatic hydrops; IT-Gd, intratympanic injection of gadopentetate dimeglumine; IV-Gd, intravenous injection of gadopentetate dimeglumine.

**Figure 4:**
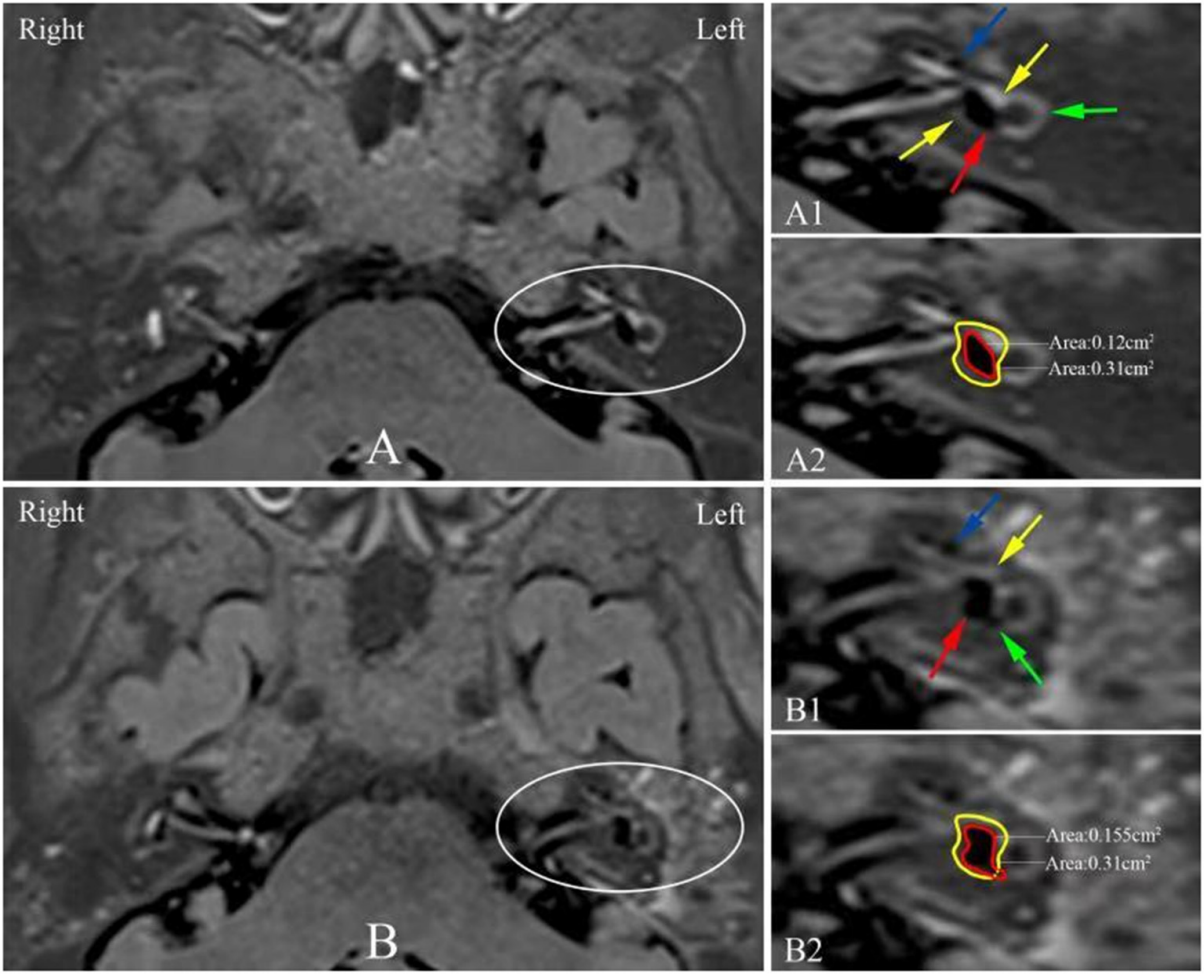
3D-real IR MRI showing a dynamic change of EH in the left ear of the patient no. 8 (Table 1) between before **(A)** and after surgery **(B)**. **(A1)** The image shows black EH (red arrow) is surrounded by white perilymph in the vestibule (yellow arrow) and a mild hydrops can be detected in the basal turn of the cochlea (blue arrow) where the lateral semicircular canal ring was visualized >240° (green arrow). **(A2)** The red solid trace indicates the endolymphatic space and the yellow solid trace indicates the fluid space in the vestibule, where the vestibular hydrops ratio (VHR) is 39%. **(B1)** Postoperative image shows the vestibular hydrops (red arrow) is increased and white perilymph space is reduced (yellow arrow), where the increased EH herniation into the non-ampullated side of LSCC (green arrow) and a significant hydrops in the basal turn of the cochlea (blue arrow) are observed. **(B2)** The red solid trace indicates the endolymphatic space and the yellow solid trace indicates the fluid space in the vestibule, where the VHR is 50%. EH, endolymphatic hydrops; LSCC, lateral semicircular canal; 3D-real IR, three-dimensional real inversion recovery; MRI, magnetic resonance imaging; MD, Meniere's disease.

Six patients showed reduction of their endolymphatic hydrops, including complete resolution of both vestibular and cochlear hydrops (Figure 2) in 2 patients, complete reversal of vestibular hydrops and partial reversal of cochlear hydrops (Figure 3) in 2 patients and reduction of vestibular hydrops but no change in cochlear hydrops in 2 patients. Thirteen patients showed their EH did not change. But three patients were found an increase in their EH (Figure 4).

The average ± standard deviation of VHR in all 22 patients was 60.41 ± 15.67 prior to surgery and 48.82 ± 18.96 following surgery. There was a significant difference in the average VHR between the values before and after surgery (*P* = 0.033).

### Correlation Between the Dynamic Changes of VHR and Vertigo Attacks

**Figure 5:**
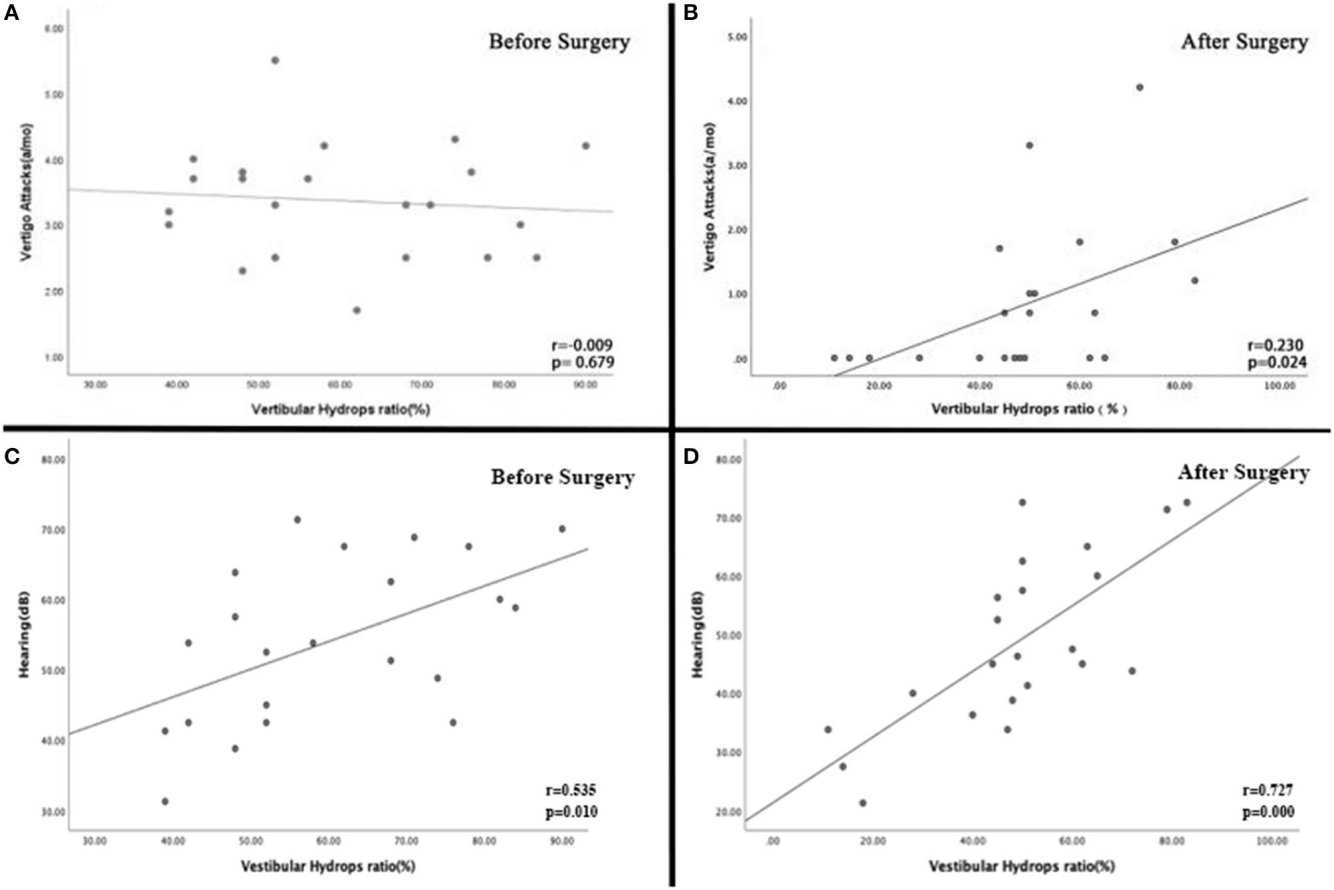
**(A)** There was no association between the number of spells prior to EDB and the preoperative VHR (*P* = 0.679). **(B)** Spearman's rank analysis showed there was a significant correlation between the number of spells in follow-up and the postoperative VHR (*P* = 0.024). **(C)** There was a significant association between the preoperative VHR and hearing threshold (*P* = 0.010). **(D)** The VHR following EDB was significantly correlated with the postoperative hearing threshold (*P* = 0.000).

Postoperatively, vertigo spells were completely controlled in 11 patients and substantially controlled in 9 patients, whereas two patients complained of their symptomatic worsening with increase in the frequency and severity of vertigo attacks. The number of spells (average ± standard deviation) prior to and following surgery was 3.41 ± 0.87 and 0.82 ± 1.15, respectively, and the difference was significant (*P* = 0.000). As shown in figures 5A and 5B, there was no association between the number of spells prior to EDB and the preoperative VHR (*P* = 0.679); however, Spearman's rank analysis showed there was a significant correlation between the number of spells in follow-up and the postoperative VHR (*P* = 0.024).

### Correlation Between the Dynamic Changes of VHR and Hearing Thresholds

All the patients (22 affected ears) were examined using the pure tone threshold audiometry before and after EDB. Hearing improved in 6 patients and fifteen patients showed no change of their hearing thresholds, whereas worsening hearing was detected in one patient. The average hearing thresholds was 54.17 ± 11.55 and 48.66 ± 14.58 (mean ± standard deviation), prior to and following EDB, respectively. There was no significant difference in the average hearing thresholds (*p* = 0.172). However, the preoperative and postoperative VHR were significantly correlated with the preoperative (Figure 5C; *P* = 0.010) and postoperative hearing thresholds (Figure 5D; *P* = 0.000), respectively.

## Discussion

In the present study, the dynamic changes in EH following EDB were reported, highlighting a potential association between the reduction of vertigo attacks, hearing preservation and decreased VHR. The use of IT-Gd + IV-Gd MRI was suggested to improve the effectiveness of imaging and evaluation techniques for EH (24) because the only IT-Gd method could provide an insufficient Gd concentration for ~10% of cases with anatomic barriers to the round window such as adhesions, bone dust blockage or thickened round window (25, 26), which could be failed to evaluate EH. No subject showed no or diagnostically insufficient inner ear contrast in our study.

The EDB procedure is reported to achieve significantly improved control of vertigo attacks compared with traditional endolymphatic sac decompression without cochlear and vestibular damage (27, 28); however, symptoms are known to fluctuate in MD, and thus cannot be used as a sufficient marker of treatment effectiveness alone. In the present study, the dynamic change of endolymphatic hydrops using Gd-enhanced inner ear MRI prior to and following EDB procedure was examined in 22 patients with intractable MD. Of these patients, 6 showed a reduction of their endolymphatic hydrops, including complete disappearance of vestibular and cochlear hydrops in 2 patients, complete reversal of vestibular hydrops and partial reversal of cochlear hydrops in 2 patients, and reduction of vestibular hydrops but no change of cochlear hydrops in the other 2 patients. Interestingly, in two severe hydropic ears, the complete reversal of hydrops was achieved in the vestibule, but not in the cochlea. This may suggest that the extreme distention of vestibular membranous structures may be reversible, whereas the severely swollen Reissner's membrane displacement into the scala vestibuli is not completely reversible, and this requires further examination.

To date, it is unknown how the degree of EH affects the patients' symptoms. Previous reports using Gd-MRI to visualize EH in patients with MD found no correlation between EH and the number of vertigo spells and high-tone hearing loss, but a significant association between low-tone and middle-tone hearing thresholds and EH (29, 30), suggesting that auditory function deteriorates with increasing EH. In the present study, there was no association between the number of spells prior to EDB and the preoperative VHR, whereas there was a significant correlation between the preoperative hearing thresholds and VHR. This finding is in agreement with the previous reports. Sepahdari et al. showed improvement of resolution of hydrops following acetazolamide therapy resulted in improvement of symptoms in patients with MD, and recurrent symptoms with recurrent hydrops after discontinuing therapy (15). Following sac surgery, hydrops was reduced, and symptoms were decreased in some cases (23), and in patients who did not respond to surgery, they showed persistence of endolymphatic hydrops (31). In the present study, 6 patients experienced reversal of symptoms and complete or partial reversal of MRI-EH, and 2 patients who complained of symptomatic worsening exhibited increased endolymphatic hydrops in the cochlea and vestibule. These results show a promising association between symptoms and hydrops. Recently, Ito et al. (32, 33) and Higashi-Shingai et al. (34) reported a positive relationship between the control of vertigo and decrease in the volume of EH and no correlation between the changes in hearing function and the volume of EH after sac drainage surgery. However, in the present study, both the postoperative average number of spells and hearing threshold were correlated with the dynamic change in postoperative VHR. This difference in hearing results could be explained by the effect of different operations because the action of EDB in treatment of MD is expected to be different from the traditional ES shunting/decompression procedures. Although an understanding of the physiologic mechanisms underlying Meniere's related vertigo and fluctuating hearing threshold is yet emerging, yet, a major body of evidence already exists that supports a direct link between hydrops and disordered auditory physiology (35) and abrupt development of hydrops was thought to play a pivotal role in the onset of vertiginous seizure (36), suggesting the therapeutic management of vertiginous seizer or acute aggravation of deafness in MD should be directed toward the control of acute development or exacerbation of EH (36), which is likely to explain why the decreased VHR was associated with reduction in the average number of spells and hearing threshold postoperatively in our study. An improvement of the average hearing threshold of 5.51 dB was also detected postoperatively, although it was not statistically significant between the before and after. Based on a suggestion by Hoa et al. (37) regarding the determining efficacy of audiometric data in treatment trials for MD, the effectiveness of a therapy may be determined by showing an increase in the percentage of stable patients >73% or increasing the percentage of fluctuators who improve audiometrically by showing an increase >9%. In the present study, hearing improved in 27.3% (6/22) of patients and stabilized in 95.5% (21/22) patients, demonstrating the effectiveness of the EDB procedure on hearing for treatment of MD. Therefore, treatments for MD focused on reducing EH were not only limited to relieving the disabling symptoms of MD, but may also be used to preserve hearing function in MD.

Notably, 13 of the 22 patients in the present study who had complete or substantial control of vertigo attacks did not exhibit changes in their hydrops following surgery, 1 patient who had substantial control of spells showed worsening of vestibular hydrops, suggesting that vertigo control may be achieved even without a reduction in hydrops. The reasons for this result are unclear. The symptomatic improvement in these patients may have been a coincidence associated with symptom fluctuation of the disease or a potential placebo effect of surgery, and will be further studied. In fact, the mechanism of vertigo attack is not completely understood and is not explained by the state of endolymphatic hydrops alone (38).

In comparison to complete or partial improvement of subjective symptoms in 90.9% of patients (20/22), the complete or partial reversal of hydrops were detected in only 27.3% of patients (6/22). Although the reversal of hydrops in the present study was far from satisfactory, these dynamic changes in hydrops provided an objective measure for verifying the effectiveness of EDB treatment in patients with MD, at least in some individuals. Thus, EDB may not be the ideal therapy for treatment of all patients with MD, only a subset. The increased hydrops was detected in 3 patients, 2 of which exhibited concomitant symptomatic worsening, suggesting that EDB does not always work to improve EH in MD. As a high degree of interindividual variability exists in the etiology of MD (11, 39), it was speculated that two etiologically distinct inner ear pathologies (hypersecretion and malabsorption of endolymph) may exist in the sacs of different patients with MD and thus not all MD patients will benefit from EDB.

The limitation of the present study is the small number of subjects and the follow-up periods of some patients were not long enough. Therefore, more cases and longer-term follow-ups are required to confirm the reversal of hydrops and symptoms following EDB, particularly for recovery of hearing, to determine whether this was due to reversal or a temporary fluctuation of the disease.

In the present study, the reversal of hydrops with a reduction in the number of vertigo attacks and hearing preservation may be used as a measure for verifying the effectiveness of EDB treatment in patients with MD.

## Data Availability Statement

The raw data supporting the conclusions of this article will be made available by the authors, without undue reservation.

## Ethics Statement

The studies involving human participants were reviewed and approved by medical ethics committee of the second Xiangya hospital of central south university. The patients/participants provided their written informed consent to participate in this study.

## Author Contributions

WJ contributions to the conception or design of the work. JHe, AP, ZZ, and YC made contributions to acquisition, analysis, or interpretation of data for the work and drafted the work. JHu and KD made the figure. QW, WL, and HC revised the content. HC provided editing and writing assistance. All authors contributed to the article and approved the submitted version.

## Conflict of Interest

The authors declare that the research was conducted in the absence of any commercial or financial relationships that could be construed as a potential conflict of interest.

## Abbreviations

EDB: endolymphatic duct blockage
MD: Meniere's disease
EH: endolymphatic hydrops
PTA: pure tone audiometry
3D-real IR: three-dimensional real inversion recovery
VH: vestibular hydrops.
